# Porphyrin Iron-Grafted Mesoporous Silica Composites for Drug Delivery, Dye Degradation and Colorimetric Detection of Hydrogen Peroxide

**DOI:** 10.1186/s11671-021-03501-6

**Published:** 2021-03-02

**Authors:** Ping Zhu, Zhihui Xu, Ling Cai, Jin Chen

**Affiliations:** 1 Center for Global Health, School of Public Health, Nanjing Medical University, Nanjing, 211166 Jiangsu China; 2 Suzhou Center for Disease Prevention and Control, Suzhou, 215000 Jiangsu China; 3 The Key Laboratory of Modern Toxicology, Ministry of Education, School of Public Health, Nanjing Medical University, Nanjing, 211166 Jiangsu China; 4 Jiangsu Province Engineering Research Center of Antibody Drug, Key Laboratory of Antibody Technique of National Health Commission, Nanjing Medical University, Nanjing, 211166 China

**Keywords:** Mesoporous silica, Interfaces, Composite materials, Hemin, Drug delivery, Real-time cell analysis

## Abstract

Porphyrin iron molecules (hemin) were successfully grafted on the channeled mesoporous silica of SBA-15 (FeIX-SBA-15), in which attached hemin molecules acted as the enzyme mimic for catalyzing oxidation reactions. In the presence of H_2_O_2_, the prepared FeIX-SBA-15 composite effectively degraded industrial dye Orange II and catalyzed tetramethylbenzidine hydrochloride (TMB) both in the solution and on the membrane, from which the colorimetric H_2_O_2_ detection was achieved. Moreover, the hemin-grafted composites showed high loading content of anticancer drug of doxorubicin hydrochloride (DOX) displaying the sustained releasing behavior as monitored by real-time cell analysis, which resulted in improved inhibitory effect on cancer cells growth compared with that DOX/SBA-15. The hemin-modified mesoporous silica nanocomposite provides an integrated nanoplatform with promising biomedical applications.

## Introduction

To overcome the disadvantages of natural enzymes such as susceptibility to denaturation under harsh environmental conditions, considerable efforts were invested to develop enzyme mimics of high stability including graphene oxide, hemin and metal nanoparticles [1, 2]. Among these artificial enzymes, hemin, the active center of heme-protein families, is a well-known natural metalloporphyrin [3]. As catalyst, metalloporphyrin complexes can effectively catalyze the oxidation of environmental pollutants like polycyclic aromatic hydrocarbons (PAHs) and azo dyes, which convert the substrate molecules into functional oxygen-containing organic compounds or degraded them to harmless compounds [4–6]. Nevertheless, the catalytic activity of hemin may suffer from the oxidative self-degradation, molecular aggregation to yield inactive dimers and low solubility in aqueous buffers [7]. Immobilization of hemin on solid support with high surface area has provided an economical yet efficient strategy to achieve its high catalytic performance while minimize the unsatisfactory loss of activity upon practical uses.

Due to the feasibility of structural adaptions to the outer and inner surfaces [8], various types of mesoporous silicon nanomaterials (MSNs) with metalloporphyrin have gained increasing attentions for diverse applications. For example, Huang et al. reported hemin-based mesoporous silica nanoreactors possessing remarkable peroxidase-like activity [9]. Barbosa et al. developed metalloporphyrins immobilized Fe_3_O_4_@SiO_2_ mesoporous submicrospheres as reusable biomimetic catalysts for hydrocarbon oxidation [10]. Very recently, Sun et al. reported a novel chemiluminescence sensor based on dual-aptamer biorecognition and hemin-encapsulated mesoporous silica for thrombin detection [11]. Among various MSNs, SBA-15 (Santa Barbara Amorphous-15) exhibits the hexagonal pore structure and adjustable pore size of 3–10 nm feasible for chemical grafting functional molecules [8, 12]. As silicon materials, SBA-15 has lower biological toxicity, and large amount of labile Si–OH groups on the surfaces of SBA-15 can be used for grafting other functional molecules to confer more functionality of SBA-15 [13]. It has been reported that SBA-15 can be used as a carrier for enzyme immobilization, antibody loading and drug delivery [14–16].

DOX as an effective chemotherapeutic antibiotic is the first-line treatment for a broad-spectrum cancer, but its side-effects in the clinics remain a serious problem [17]. To improve the therapeutic efficacy while to decrease the systematic toxicity of DOX, considerable efforts have been made on molecular design as well as formulation development of various drug delivery systems. After the mesoporous MCM-41 (Mobil Composition of Matter No. 41) was firstly used as drug carrier in 2001 [18], MSNs including SBA-15 possessed advantageous feature [19, 20] because of their inherent pore structure desirable for drug loading and release. Nevertheless, the complicated chemical modifications on MSNs may limit their practical application.

**Scheme 1.**
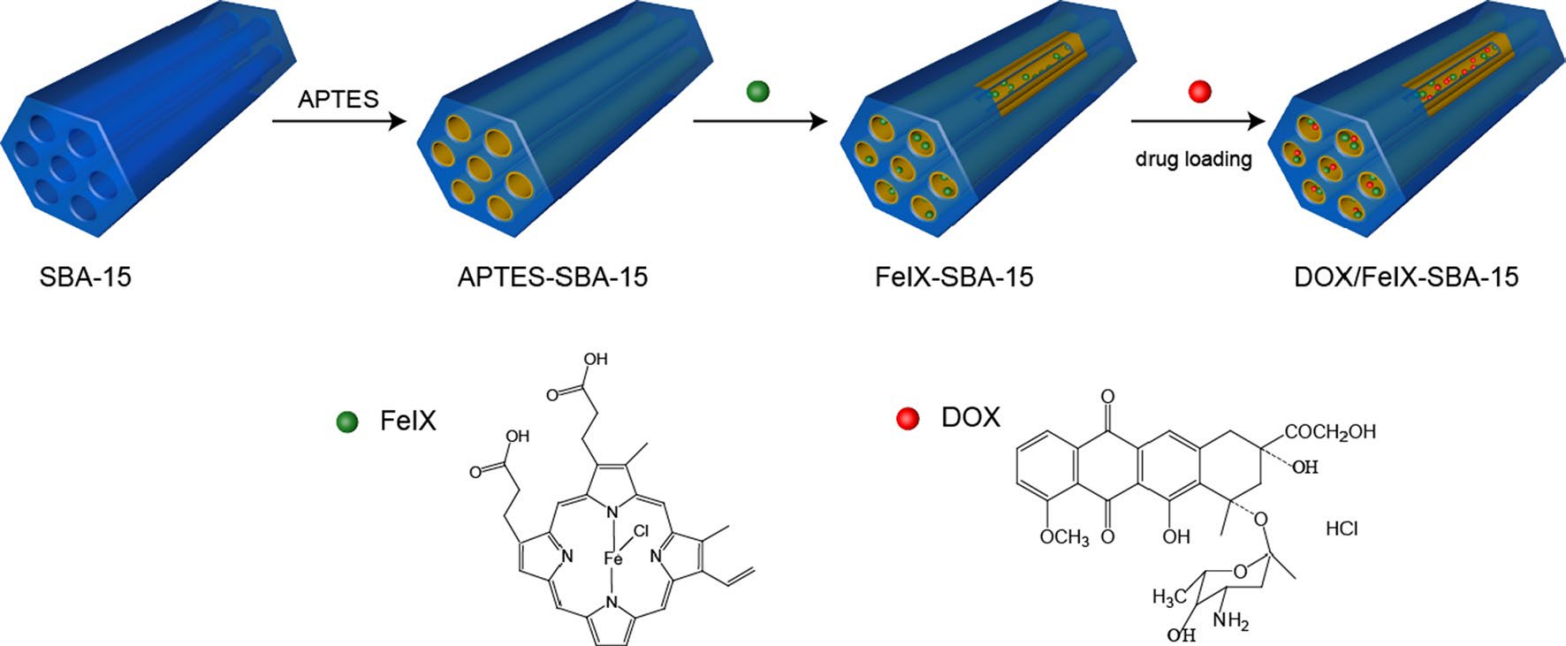
The SBA-15 grafted with FeIX for anticancer drug DOX loading

In this study, we successfully grafted hemin on the SBA-15 to build a composite material (FeIX-SBA-15) (Scheme 1), in which not only the enzyme-like activity of hemin was retained, but the efficient encapsulation and sustained release of doxorubicin hydrochloride (DOX) were achieved as reflected by the growth curve of incubated cancer cells by the real-time cell analyzer (RTCA) technology [21–23]. Notably, a relatively high loading content of DOX was obtained for DOX/FeIX-SBA-15 as compared with our earlier work using ferrocenecarboxylic acid-modified SBA-15 (FCA-SBA-15) [24] which may be due to the refined π-π stacking between grafted FeIX and DOX in the support. In addition, owing to the solid form of FeIX-SBA-15 that was immobilized on a commercial filter membrane, a flow catalysis format for effective dye degradation and colorimetric detection of hydrogen peroxide was developed.

## Materials and Methods

### Materials

All reagents were of analytical grade (A.R.) and used without further purifications. Tri-block copolymer Pluronic P123 (EO_20_PO_20_EO_20_, MW = 5800) was purchased from Sigma-Aldrich (Germany). 3-aminopropyltriethoxysilane (APTES), tetraethylorthosilicate (TEOS), acid orange II and tetramethylbenzidine hydrochloride were obtained from Shanghai Aladdin biological technology Co., Ltd (China). Hemin and doxorubicin hydrochloride were purchased from Shanghai Macklin Biochemical Co., Ltd (China). Trypsin–EDTA solution was obtained from Beyotime Biotechnology Co., Ltd (China). Cell culture media (RPMI-1640) were from GE Healthcare Life Sciences Co., Ltd (China). Fetal bovine serum (FBS) was obtained from Gibco Co., Ltd (USA). Penicillin and streptomycin were from Thermo Fisher Scientific Co., Ltd. The A549 cell line was obtained from the American Type Culture Collection (ATCC).

### Chemical Grafting of SBA-15 for Drug Loading

SBA-15 was prepared as previously reported [12]. Subsequently, 0.40 g SBA-15 was dispersed in 140 mL methylbenzene at 80 °C and APTES (1.2 mL) was added. Then, the mixture was stirred for another 8 h and separated by centrifuging at 5000 rpm for 5 min. After washing with ethanol and water, the resulting products that APTES-SBA-15 were dried at 80 °C. Hemin (0.15 g) was firstly dispersed in 30 mL DMSO and followed by the addition of APTES-SBA-15 (0.60 g), and then the mixture was stirred for another 7 h at 70 °C. The resulting product was centrifuged, washed and finally dried, which was FeIX-SBA-15.

After FeIX was validated to be successfully grafted onto SBA-15, and FeIX-SBA-15 (0.50 g) was suspended in 20 mL of deionized water containing DOX·HCl (2 mg/mL) stirring at 37 °C for 24 h to load DOX. Then, the products were centrifuged at 5000 rpm for 5 min. After washing, drying and grinding, the final products were collected (DOX/FeIX-SBA-15).

### Characterizations

The morphological features of the sample were studied by scanning electron microscope (SEM, Hitachi SU-1510) with energy-dispersive spectroscopy (EDS) X-ray detector operated at an accelerating voltage of 15 kV. The small angle X-ray diffraction (SAXRD) patterns of prepared materials were collected by a Smartlab TM 9 KW X-ray diffractometer using Cu Kα radiation (*λ* = 0.154 nm) in the 2θ of 0.2°–8°. X-ray fluorescence (XRF) analysis was measured on X-ray fluorescence spectrometer (Thermo Scientific, USA). The nitrogen sorption isotherms were measured on a volumetric adsorption analyzer (BELSORP-MINI, Japan) in a relative pressure range P/P_0_ from 0.01 to 0.99. The specific surface area and distribution of pore size were calculated using the Brunauer–Emmett–Teller (BET) and Barrett–Joyner–Halenda (BJH) measurements, respectively. Solid UV–vis spectra of materials were recorded by Solid UV–vis spectrophotometer (Thermo Scientific, USA). The absorption spectra of samples were measured on an ultraviolet and visible spectrophotometer (UV–vis 7300, China) to calculate DOX drug loading content (DLC) according to the following formula: DLC (wt. %) = (weight of loaded drug/total weight of mesoporous material and loaded drug) *100%. The iron content of DOX/FeIX-SBA-15 was determined by an Inductively Coupling Plasma emission spectrometer (ICP, PE Optima 2000DV, USA). Before analysis, the DOX/FeIX-SBA-15 was completely dissolved in hydrofluoric acid firstly, then hydrofluoric acid was volatilized and the sample was re-dissolved in concentrated nitric acid.

### Catalytic Activity and Degradation Behaviors of FeIX-SBA-15

Taking advantage of the enzyme-like activity of FeIX, the catalytic activity of the TMB and the degradation behaviors of the acidic orange II in solution were investigated. A mixed solution containing 20 mM NaOH and 1.5 mM Triton X-100 was prepared, and then, it was diluted 4 times with PB solution for dissolving the FeIX-SBA-15. In the TMB catalytic reaction, 500 μL of FeIX-SBA-15 (600 μg/mL) mixed with 500 μL of H_2_O_2_ (2 mM) as substrate was used to degrade TMB (500 μL, 3 mg/mL). The spectral measurements were carried out at specific time intervals to evaluate the degree of reaction. In order to study the degradation behaviors of synthesized composites, the mixture of 500 μL of FeIX-SBA-15 (600 μg/mL) solution and 500 μL of 10 mM H_2_O_2_ solution served as substrate. Next, 500 μL of Orange II (0.25 mM) was added into above solution. The absorbance measurements were recorded at 485 nm.

Additionally, the composites were further immobilized on the commercial filter membrane to degrade acid orange II in a flow catalysis manner. 5 mL suspended solution of FeIX-SBA-15 (600 μg/mL) was passed through a commercial filter (0.22 μm, Millipore) to allow the material to be trapped on the filter and dried at the room temperature. 500 μL of Orange II (0.25 mM) solution mixed with 500 μL of H_2_O_2_ (10 mM) and 500 μL H_2_O was passed through the commercial filter membrane loaded with FeIX-SBA-15. The spectral measurements of the mixture were recorded.

### Colorimetric Detection of H_2_O_2_

For H_2_O_2_ detections in solution, the mixtures of 500 μL of FeIX-SBA-15 (600 μg/mL) and 500 μL TMB (3 mg/mL) were prepared. Next, 500 μL of H_2_O_2_ of varying concentrations (25–500 μM) was added to the above solutions and incubated for 10 min at 30 °C. Finally, the spectral measurements were recorded at 651 nm.

Simultaneously, the measurement of H_2_O_2_ was conducted on the commercial filter membrane immobilized the composites in a flow catalysis manner. The modified membrane was obtained as described above. Then, the mixtures of 500 μL of H_2_O_2_ (0.293 ~ 8.8 mM), 500 μL TMB (2 mg/mL) and 500 μL H_2_O were passed through the modified commercial filter membranes separately, after which the spectral measurements of the mixture were recorded at 651 nm.

The absorbance was plotted against the concentration of H_2_O_2_, and the detection limit (LOD) of the method is evaluated via the formula: LOD = 3RSD/slope. (RSD: relative standard deviation).

### Cell Clture and RTCA Detection

Human non-small cell lung cells A549 were cultured with RPMI-1640 medium, containing 10% FBS, 1% penicillin–streptomycin solution in an incubator containing 5% CO_2_ at 37 °C (Thermo Scientific). Real-time cell analyzer (RTCA) technology (xCELLigence system, ACEA Biosciences Inc.) and Cell Counting Kit-8 (CCK-8, DOJINDO Laboratory) method were employed for the cytotoxicity evaluation of the materials. In RTCA detection, 5000–8000 cells per well were seeded in E-plate. Cell incubation and proliferation were monitored on a real-time basis by the analyzer, in which the signal changes were expressed as an arbitrary unit defined as cell index (CI). Cells were exposed to FeIX-SBA-15 and DOX/FeIX-SBA-15 at a concentration of 12.6 µg/mL. The concentration of DOX was 0.50 µg/mL. Besides, to obtain the IC_50_ of DOX/FeIX-SBA-15, cells treated with different doses of DOX/FeIX-SBA-15 from 1.6 to 50.4 µg/mL were detected. In addition, CCK-8 kit assay as an endpoint method was used to detect the cytotoxicity of materials. The CCK-8 reagent incubated with cells for 2 h and the absorbance was measured by microplate reader at the wavelength of 450 nm.

## Results and Discussion

### Materials Characterizations

The composite of DOX/FeIX-SBA-15 was synthesized as illustrated in Scheme 1. APTES-SBA-15 was prepared firstly via amination reaction [13]. Then, the molecules of FeIX were grafted on the surface of APTES-SBA-15 through an amide reaction and electrostatic interaction between the carboxyl groups and the amino groups in the mesopore. Finally, the anticancer drug DOX loaded into FeIX-SBA-15 composites involving the strong molecular interactions of π-π stacking between FeIX and DOX due to the conjugated planar macrocyclic molecule of FeIX [25] and the anthracycline chromophore of DOX [24].

**Fig. 1:**
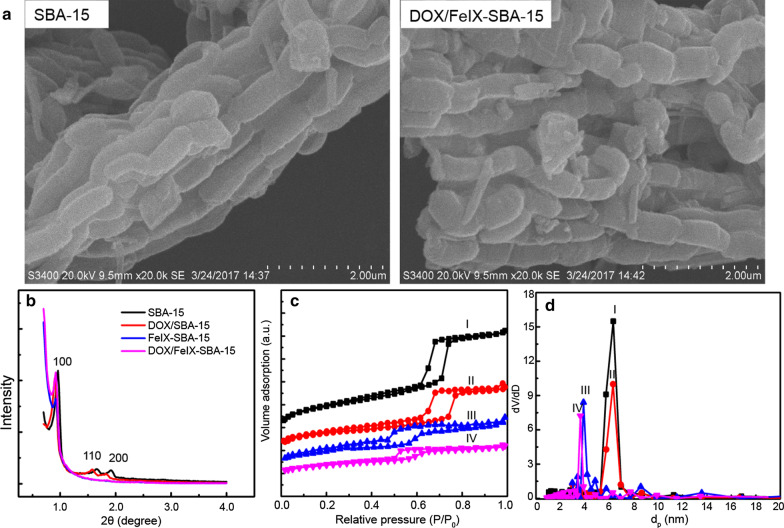
**a** SEM images of SBA-15 and DOX/FeIX-SBA-15. **b** Small angel X-ray diffraction patterns of materials. Nitrogen adsorption isotherms (**c**) and pore size (**d**) of obtained materials: **I** SBA-15, **II** DOX/SBA-15, **III** FeIX-SBA-15 and **IV** DOX/FeIX-SBA-15

The composites surface microstructure was evaluated by scanning electron microscopy (SEM). As shown in Fig. 1a, SBA-15 of tubular structures with certain uniformity of size of 0.4–1 μm were formed in SBA-15 and attached FeIX and DOX molecules caused no apparent morphological changes. The TEM images (Fig. S1) of DOX/FeIX-SBA-15 in comparison with SBA-15 validated the retained mesostructure after the chemical modifications. The chemical composition of DOX/FeIX-SBA-15 was further estimated by X-ray fluorescence spectroscopy. As shown in Table S1, Si, O, C, Fe and trace amount of other absorbed elements were found in DOX/FeIX-SBA-15. Compared with that of FeIX-SBA-15, the calculated atom percentage of Si (~ 24.3%) and Fe (~ 2.5%) in DOX/FeIX-SBA-15 were slightly lower, but the amount of C (11.3%) is relatively high suggesting the successful encapsulation of DOX. To further evaluate the surface components, solid UV–vis spectra of composites were recorded. As shown in Fig. S2, similar to that of FeIX molecules, FeIX-SBA-15 displayed absorption bands at 250–350 nm, 450–550 nm and 600–700 nm, while there were no virtually observable bands for SBA-15 [26]. By comparison, DOX/FeIX-SBA-15 exhibited a wide absorption band of 450–550 nm arising from the DOX.

The small angle X-ray diffraction analysis of composites was also conducted. As shown in Fig. 1b, the SAXRD patterns of SBA-15 displayed a main diffraction peak at 0.94° with two observable peaks at 1.6° and 1.8° reflecting (100), (110) and (200) crystalline planes, respectively, pointing to a well-defined mesostructure [27]. Compared to SBA-15, the SAXRD patterns of DOX/SBA-15 showed a decrease in peak intensity, indicating that the loading DOX brought no damage to the pore structure. However, grafting FeIX in SBA-15 resulted in an observable peak shift towards large angles with decreased peak intensity of (110) and (200) crystalline planes suggesting the partial loss of mesostructural regularity of materials [28]. Notably, further loading of DOX in FeIX-SBA-15 caused the disappearance of (110) and (200) crystalline planes.

To obtain the mesostructural parameters of the samples, the nitrogen sorption isotherms were recorded. As shown in Fig. 1C, all the samples exhibited typical type IV isotherms with a sharp capillary condensation step at high relative pressures, indicating the retaining of mesostructure after chemical modifications [24]. As shown in Fig. 1D and Table S2, compared with that of SBA-15 of ~ 6 nm, a decrease in pore size upon conjugation of FeIX/DOX was observed corroborating the results of SAXRD. The DOX loading content in DOX/SBA-15 and DOX/FeIX-SBA-15 was calculated to be 1.14% and 4.27%, respectively. The results indicated that FeIX-grafted SBA-15 enhanced the load capability of DOX in the mesopores which was ~ 3.7-fold of DOX/SBA-15 and ~ 1.6-fold of that of DOX/FCA-SBA-15 from our earlier work [24]. Such improved loading capability of drug molecules could be ascribed to the refined molecular interactions between FeIX and DOX.

### Catalytic Activity and Degradation Behaviors of Grated Hemin on Mesopores

**Fig. 2:**
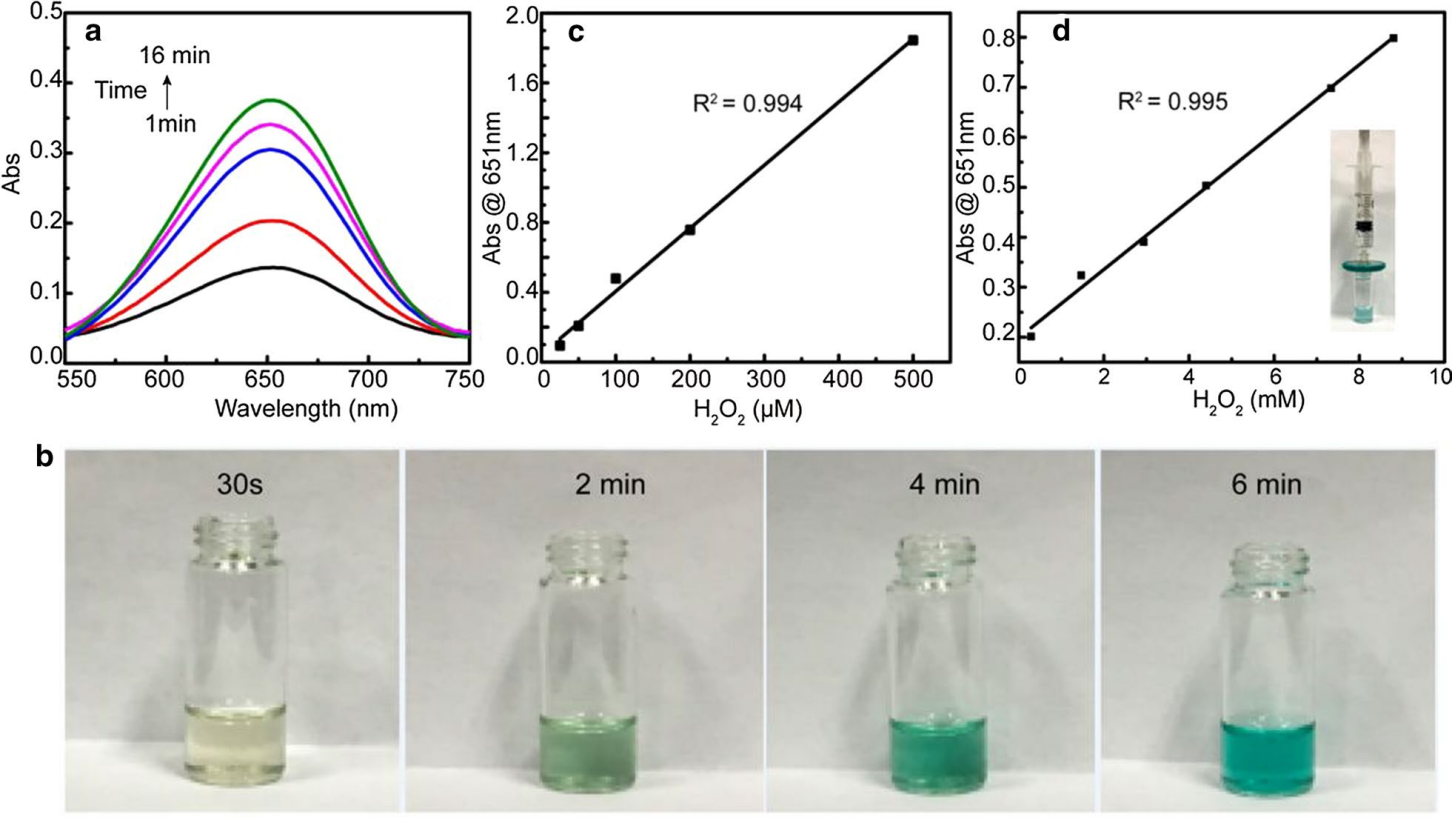
**a** The UV–vis absorbance change of FeIX-SBA-15 mixed with TMB and H_2_O_2_. **b** Photographs of solutions containing TMB, H_2_O_2_, and FeIX-SBA-15 at different time. The linear calibration plot of H_2_O_2_ in solution (**c**) and on FeIX-SBA-15 modified membrane (**d**)

The catalytic activity of FeIX-SBA-15 was evaluated by using TMB in the presence of H_2_O_2_ as a model reaction [29]. Figure 2a showed that the absorbance intensity of assay solution (TMB + H_2_O_2_ + FeIX-SBA-15) increased with the catalytic reaction time. Accordingly, solutions changed to blue color and were getting darker with time (Fig. 2b). However, the reaction did not occur when either H_2_O_2_ or FeIX-SBA-15 was absent in the solution indicating a peroxidase activity of FeIX-SBA-15. Meanwhile, as shown in Fig. S3A, FeIX-SBA-15 was able to degrade Orange II in the presence of H_2_O_2_ as monitored by the UV–vis absorbance within 3-h reaction time.

Taking advantage of solid form of hemin-graft mesoporous composites, a flow catalysis format based on the FeIX-SBA-15-immobilized commercial filter membrane was tested for organic transformations [30, 31]. As shown in Fig. S3B, when H_2_O_2_ and Orange II mixture passed through the modified membrane, compared to the control membrane with SBA-15 only (Fig. S3C), the absorption intensity of solution decreased concomitantly indicating the FeIX-SBA-15 immobilized membrane of great catalytic activity after reuse, which could be applied in the dye degradation in wastewater [32, 33].

Additionally, compared with horseradish peroxidase, composites containing hemin exhibited remarkable catalytic activity in the wide range of pH attributing to sufficient stability of hemin under relatively harsh conditions including acidic solution [29, 34], which is of practical importance.

### Colorimetric Detection of H_2_O_2_

Based on the TMB catalysis reaction model of FeIX-SBA-15, a colorimetric strategy for the determination of H_2_O_2_ in solution was established with the calibration plot shown in Fig. 2c. The concentration range of H_2_O_2_ was of 25–500 μM with a detection limit (LOD) of 2.1 μM.

Sequentially, a simple chromogenic detection of H_2_O_2_ was also developed by direct filtering of H_2_O_2_ of varied concentrations through the FeIX-SBA-15 modified commercial membrane. As shown in Fig. 2d, the linear detection range is estimated to be from 0.293 to 8.80 mM with a detection limit of 0.067 mM. The comparison of the analytical parameters obtained with those of earlier reports is tabulated in Table 1, which is indicative of detection performance related to the reaction conditions such as catalyst concentration, pH and assay temperature [35]. Although the proposed method did not outperform those earlier reports, its analytical performance with a broad calibration concentration range was comparable with chromogenic methods.

**Table 1:** The analytical parameters of reported Fe-based catalysts for H_2_O_2_ detection

Sample	Liner range (µM)	LOD (µM)	References
Fe-SBA-15	0.4–15	0.2	[36]
Fe-SAzyme	10–150	1.8	[37]
CSA-hemin NPs	5–400	3.32	[29]
hemin-GroEL	0–300	10	[38]
Fe_3_O_4_@CP	0.2–300	0.11	[39]
Fe_3_O_4_@SiO_2_@Au MNPs	1–40	0.6	[40]
FeIX-SBA-15 (in solution)	25–500	2.1	This work
FeIX-SBA-15 (on membrane)	0.293–8.8 mM	0.067 mM	This work

### Cytotoxicity Assay and Dynamic Monitoring the Effects of DOX-Loaded Complexes on Cells

**Fig. 3:**
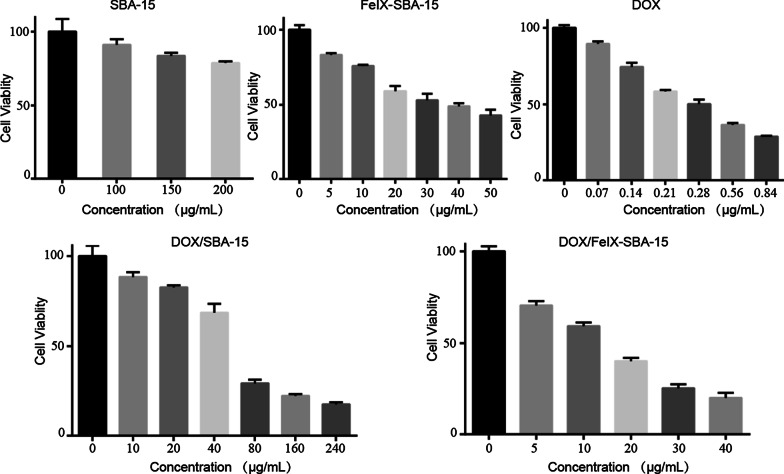
Viability of A549 cells incubated with SBA-15, FeIX-SBA-15, DOX, DOX/SBA-15, DOX/FeIX-SBA-15, respectively

As shown in Fig. 3, the growth inhibitory effects of samples on A549 cells were firstly evaluated by CCK-8 kit and the measured half-inhibitory concentrations (IC_50_) of 24 h are summarized in Table S3. The cells treated by SBA-15 of 150 μg/mL still retained more than 80% cell viability reflecting the low cytotoxicity of materials. The IC_50_ of DOX/FeIX-SBA-15 (12.6 μg/mL) was ~ fourfold lower than that of DOX/SBA-15 (58.8 μg/mL) and ~ threefold lower than FeIX-SBA-15 (35.4 μg/mL), which suggested the grafting of FeIX on SBA-15 was efficient to load DOX to improve the cytotoxic effect.

**Fig. 4:**
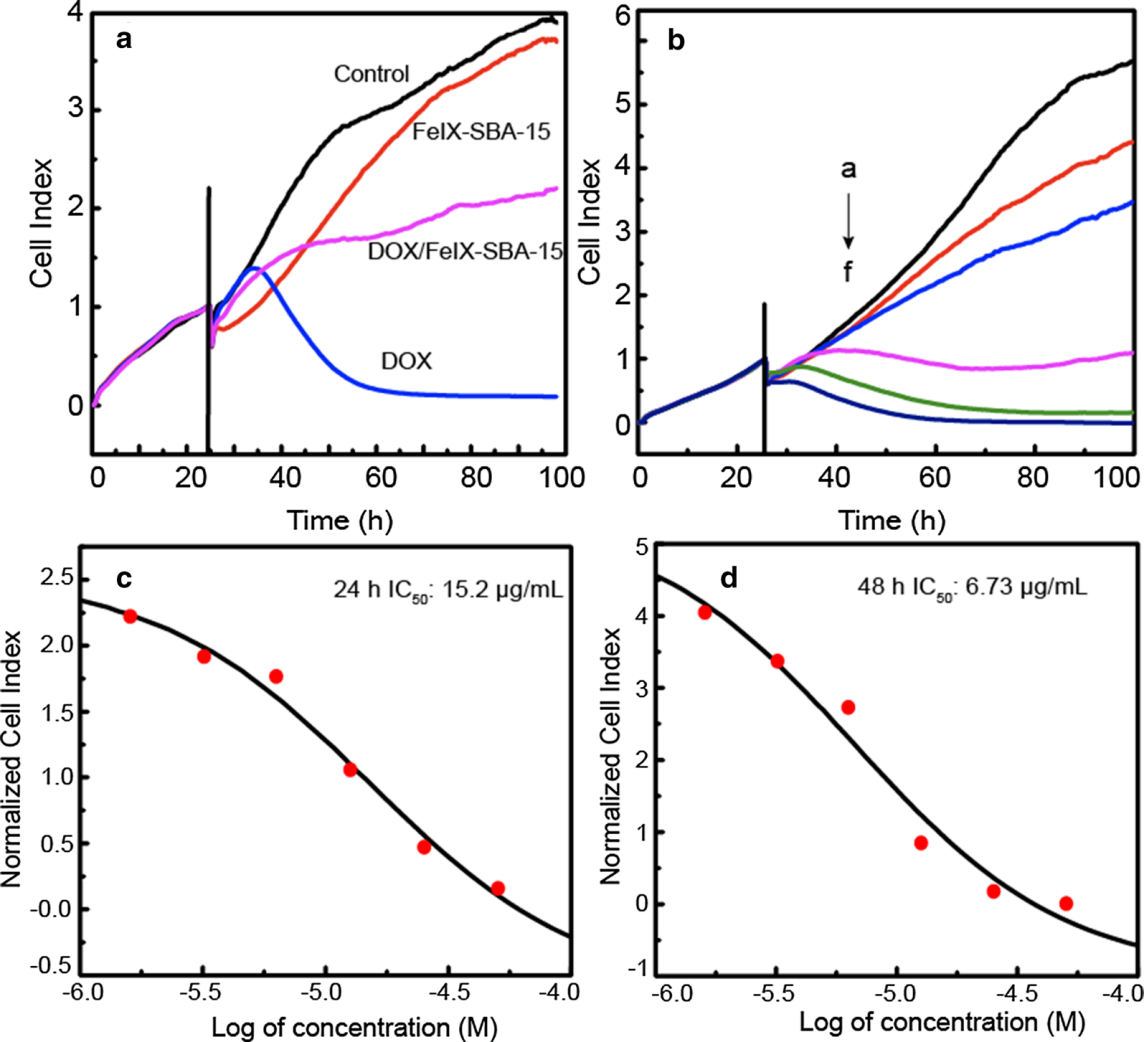
The cell growth of A549 cells treated with different materials (**a**) and (**b**) different concentrations of DOX/FeIX-SBA-15 (a-f: 1.6, 3.2, 6.3, 12.6, 25.2 and 50.4 µg/mL) with the dose–response curves (**c**, **d**). The black line in Fig. **a**&**b** suggests the time point of adding materials

The growth statuses of A549 cells treated with different composites showed the sustained drug release in Fig. 4a, b monitored by RTCA, which is based on a label-free impedance detection principle to reflect the physiological conditions of cells [41]. As shown in Fig. 4a, at the tested dosage, the cell index values of DOX treated-cells increased firstly and subsequently decreased rapidly, a sharp decrease in normalized cell index (NCI) values observed involving a DNA damaging process [42]. The NCI values of both FeIX-SBA-15 (12.6 μg/mL) and DOX/FeIX-SBA-15 (12.6 μg/mL) were lower than the control, but the NCI values were observed to increase with the incubation time. Compared with FeIX-SBA-15, the NCI values of DOX/FeIX-SBA-15 treated-cells increased during the first several hours of treatment. However, due to the sustainable release behaviors of drug delivery and the effective concentration accumulation of DOX released from the mesopores of DOX/FeIX-SBA-15 was not enough to kill the most of cells, the cell growth of A549 cells treated by DOX/FeIX-SBA-15 exhibited a relatively stable and inhibitory state, while the cell index values of FeIX-SBA-15 treated-cells increased significantly in the next process. At the IC_50_ concentration of 12.6 μg/mL (CCK-8), the NCI value of DOX/FeIX-SBA-15 was found higher than the 50% of the control group at 24 h. Therefore, a multiple-dose effect of DOX/FeIX-SBA-15 on cells was recorded (Fig. 4B) and the derived dose–response curves from the recorded NCI of DOX/FeIX-SBA-15 were calculated using RTCA software (Fig. 4c, d). The IC_50_ value of DOX/FeIX-SBA-15 treated cells was determined to be 15.0 (24 h) which was consistent with CCK-8 tests. And after 48 h-incubation, the IC_50_ calculated was 6.7 (48 h) µg/mL.

## Conclusion

In this study, we successfully grafted hemin molecules on SBA-15 for multiple uses. The constructed FeIX-SBA-15 was desirable for loading anticancer drugs and was effective to catalyze TMB and degrade acid orange II both in solution and on the membrane in the presence of H_2_O_2_. On the basis of TMB catalysis model reaction, a colorimetric strategy for the quantitative analysis of H_2_O_2_ was established. Additionally, the FeIX-grafted SBA-15 favored a relatively high loading content of DOX and improved inhibitory effect on the cancer cell growth compared with that of DOX/SBA-15. Meanwhile, the cytotoxicity of DOX/FeIX-SBA-15 on A549 was dynamically monitored by RTCA, evidently suggesting sustained-release behaviors of drug molecules DOX from mesopores. On this basis, this drug delivery system reduced the cytotoxicity of DOX but was still effective in inhibiting the growth of tumor cells. Taken together, the hemin-grafted mesoporous silica nanocomposite we produced as solid catalyst and drug delivery system could provide a versatile nanoplatform with enormous biomedical potentials.

## Supplementary Information

**Additional file 1**.

## Data Availability

All data are fully available without restriction.
