# Supplementary material for: Porphyrin Iron-Grafted Mesoporous Silica Composites for Drug Delivery, Dye Degradation and Colorimetric Detection of Hydrogen Peroxide
Source: Nanoscale Res Lett. 2021 Mar 2;16:41. doi: 10.1186/s11671-021-03501-6 (PMC7925758; doi:10.1186/s11671-021-03501-6)

**Supporting information**

**Porphyrin iron grafted mesoporous silica composites for drug delivery, dye degradation and colorimetric detection of hydrogen peroxide**

Ping Zhu^1#^, Zhihui Xu^2,3#^, Ling Cai^1^, Jin Chen^1,3*^

^1^Center for Global Health, School of Public Health, Nanjing Medical University, Nanjing 211166, Jiangsu, China

^2^Suzhou Center for Disease Prevention and Control, Suzhou 215000, Jiangsu, China

^3^The Key Laboratory of Modern Toxicology, Ministry of Education, School of Public Health, Nanjing Medical University, Nanjing 211166, Jiangsu, China

^#^Equal contribution.

*Correspondence: jchen@njmu.edu.cn; okachen30@gmail.com

**Table S1.** Element estimation of FeIX-SBA-15 and DOX/FeIX-SBA-15 by quantitative XRF analysis

| Sample | Element | Value | unit | Limit of detection | El. line | Intensity | w/o normal |
| --- | --- | --- | --- | --- | --- | --- | --- |
| FeIX-SBA-15 | Si | 26.5 | mass % | 0.006 | Si | 26.5 | mass % |
|  | Fe | 2.6 | mass % | 0.002 | Fe | 2.6 | mass % |
|  | C | 9.8 | mass % | 0.156 | C | 9.8 | mass % |
| DOX/FeIX-SBA-15 | Si | 24.3 | mass % | 0.005 | Si | 24.3 | mass % |
|  | Fe | 2.5 | mass % | 0.001 | Fe | 2.5 | mass % |
|  | C | 11.3 | mass % | 0.155 | C | 11.3 | mass % |

**Table S2.** Mesoporous parameters of obtained composites

| sample | D_p_ / nm | pore volume / cm^3^·g^-1^ |
| --- | --- | --- |
| SBA-15 | 6.327 | 1.557 |
| DOX/SBA-15 | 6.310 | 0.991 |
| FeIX-SBA-15 | 3.997 | 0.712 |
| DOX/FeIX-SBA-15 | 3.640 | 0.504 |

**Table S3.** IC_50_ (24 h) determined by CCK-8

| sample | IC_50_ (µg/mL) |
| --- | --- |
| FeIX-SBA-15 | 35.4 |
| DOX | 0.332 |
| DOX/SBA-15 | 58.8 |
| DOX/FeIX-SBA-15 | 12.6 |

**Figure S1**. TEM images of (A) SBA-15 and (B) DOX/FeIX-SBA-15.


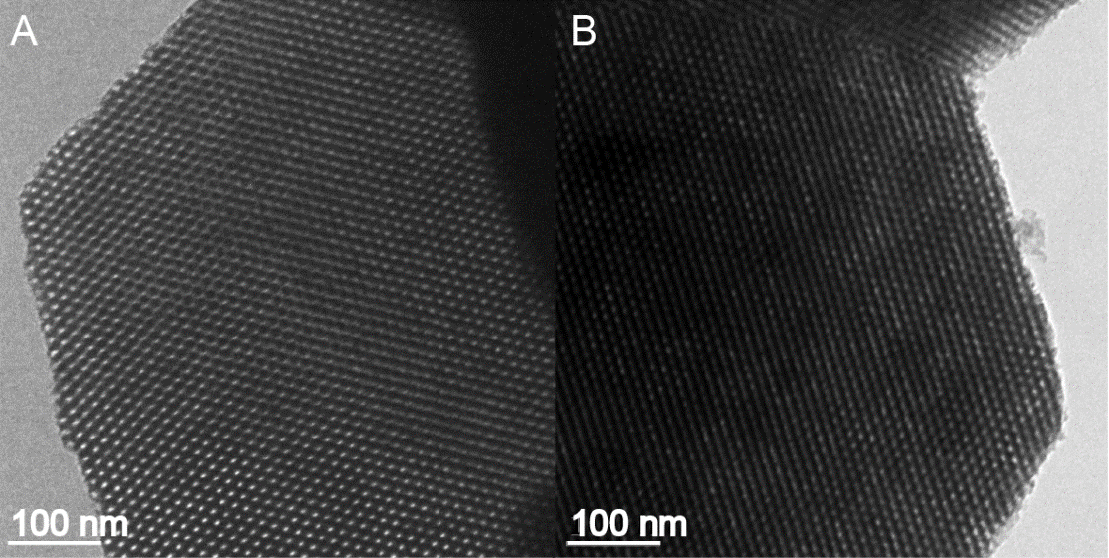


**Figure S2**. Solid UV-vis spectra of materials.


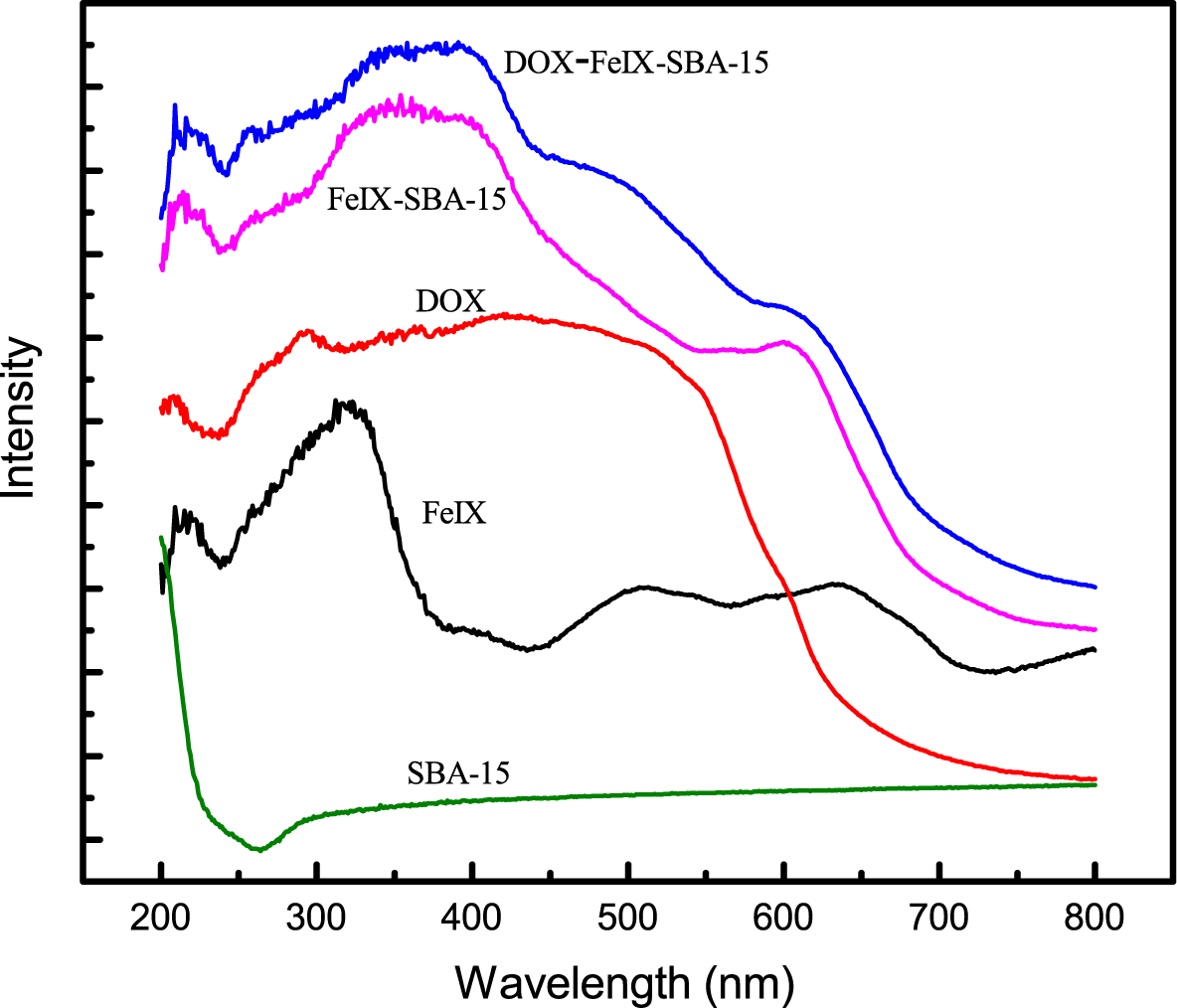


**Figure S3.** The UV spectra of changes in the maximum absorbance at 485 nm for Orange II with the time (A) and the frequency (B) of oxidation catalyzed by FeIX-SBA-15 complex. (C) The pattern of the absorbance changes at 485 nm with the frequency of passing through membrane.


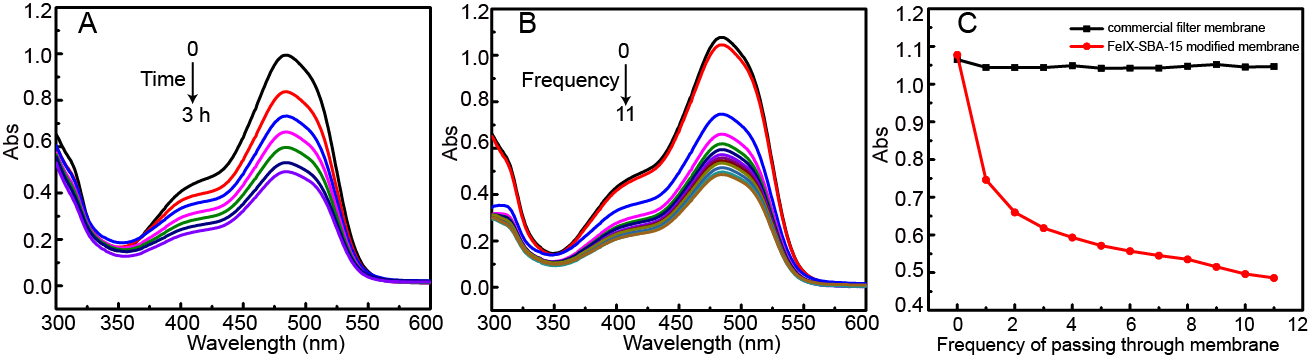

Supplement: Supplementary file 1 — Additional file 1. [file 11671_2021_3501_MOESM1_ESM.docx]
